# American Medical Association, May, 1888

**Published:** 1888-04

**Authors:** 


### AMERICAN MEDICAL ASSOCIATION, MAY, 1888.

Members of the Association will be pleased to learn that the organization of the Committee of Arrangements for the entertainment of the American Medical Association in Cincinnati in May, has been completed, and that long strides have been taken towards securing for the Congress scientific work of high order and social features that will be at least acceptable. Dr. W. W. Dawson, the Chairman of the Committee, has associated with himself a number of representative medical men to aid him in the work of the Committee. To facilitate this work the following Sub-committees and Chairmen have been appointed.

| *Sub-committee.* | *Chairman.* |
|---|---|
| 1. Section-Work | J. T. Whittaker. |
| 2. Halls and Decorations | B. Stanton. |
| 3. Entertainments | N. P. Dandridge. |
| 4. Exhibits | J. C. Culbertson. |
| 5. Programme | J. Ransohoff. |
| 6. Reception | J. T. Whittaker. |
| 7. Finance | S. C. Ayres. |
| 8. Registration | Jas. M. French. |
| 9. Printing and Invitations | J. C. Culbertson. |
| 10. Transportation and Hotel | Geo. Purviance. |

Music Hall has been secured for the second week in May. Under its vast roof the various Sections of the Association can be easily accommodated. The seating capacity of the rooms for Section-Work varies from about 70 to 300. Their acoustic properties approach perfection.

